# Long‐term consequences of early postnatal lead exposure on hippocampal synaptic activity in adult mice

**DOI:** 10.1002/brb3.1307

**Published:** 2019-07-03

**Authors:** Anahis Tena, Eduardo Peru, Luis E. Martinetti, Jose C. Cano, Carla D. Loyola Baltazar, Amy E. Wagler, Rachid Skouta, Karine Fenelon

**Affiliations:** ^1^ Department of Biological Sciences, College of Science University of Texas at El Paso El Paso Texas; ^2^ Department of Mathematical Sciences, College of Science University of Texas at El Paso El Paso Texas; ^3^ Department of Chemistry, College of Natural Science University of Massachusetts Amherst Amherst Massachusetts; ^4^ Biology Department, College of Natural Science University of Massachusetts Amherst Amherst Massachusetts

**Keywords:** lead exposure, in vitro electrophysiology, hippocampus, prefrontal cortex, slices, optogenetics

## Abstract

**Introduction:**

Lead (Pb) exposure yielding blood lead levels (BLL) as low as 2 µg/dl in children is an international problem. More common in US low‐income neighborhoods, childhood Pb exposure can cause behavioral and cognitive deficits, including working memory impairments, which can persist into adulthood. So far, studies characterized short‐term effects of high Pb exposure on neuronal structure and function. However, long‐term consequences of early chronic Pb exposure on neuronal activity are poorly documented.

**Methods:**

Here, we exposed male and female mice (PND [postnatal day] 0 to PND 28) to one of three Pb treatments: 0 ppm (sodium‐treated water, control), 30 ppm (low dose), and 330 ppm (high dose) lead acetate. Once the male and female mice were 9–12 months old, extracellular field recordings on hippocampal slices were performed.

**Results:**

We show that at CA3 to CA1 synapses, synaptic transmission was decreased and neuronal fiber activity was increased in males exposed to lowest level Pb. In contrast, both synaptic transmission and neuronal fiber activity were increased in females exposed to high Pb. The ventral hippocampus–medial prefrontal cortex (vHPC–mPFC) synapses are crucial for working memory in rodents. The lowest level Pb decreased vHPC–mPFC synaptic transmission, whereas high Pb decreased short‐term synaptic depression.

**Conclusions:**

Overall, we show for the first time that early exposure to either high or lowest level Pb has long‐term consequences on different synaptic properties of at least two hippocampal synapses. Such consequences of early Pb exposure might worsen the cognitive decline observed in aging men and women. Our results suggest that additional efforts should focus on the consequences of early Pb exposure especially in at‐risk communities.

## 1 INTRODUCTION

Lead (Pb) is a pervasive environmental neurotoxicant that can cause developmental, motor, behavioral, and cognitive deficits in humans (Bellinger, 2008a, 2008b; Lanphear, Hornung, & Ho, 2005a, 2005b; Needleman, 2004; Zhang et al., 2013). Such Pb‐induced deficits are more common in the low‐income neighborhoods found throughout the United States (Bernard & McGeehin, 2003; Rios‐Arana, Walsh, & Gardea‐Torresdey, 2004; Rothman, Lourie, & Gaughan, 2002). This is partly because children from these at‐risk communities are exposed early in life to Pb levels considered nontoxic. Unfortunately, even low Pb levels can result in impairments in hippocampus‐dependent learning and memory, attention deficits, and reduced intelligence quotient (IQ) scores (Bellinger & Needleman, 2003; Needleman & Gatsonis, 1990; Needleman & Landrigan, 2004; Lanphear, Dietrich, Auinger, & Cox, 2000; Lanphear, Hornung, & Ho, 2005a, 2005b). So far, studies have focused on the neuronal effects of high Pb levels yielding blood lead levels (BLLs) greater than 10 µg/dl. However, to date, no safe (nontoxic) BLL has been identified (Reiley, 2007). For example, BLLs as low as 2 µg/dl were associated with decreased cognitive functions and lower academic success in children (Huang et al., 2012; Lucchini et al., 2012; Sobin, Flores‐Montoya, Gutierrez, Parisi, & Schaub, 2015). Other studies show that some of these childhood deficits can persist long after Pb exposure has ceased (Barkur, Rao, & Bairy, 2011; Gilbert & Lasley, 2002; Schwartz et al., 2000). Data from adults in their fifties (equivalent to 9‐ to 12‐month‐old mice) suggest that Pb exposure can accelerate cognitive decline (Mason, Harp, & Han, 2014) and worsen the memory impairment of Parkinson's and Alzheimer's disease patients (Eid & Zawia, 2016; Lee & Freeman, 2014). In humans, Pb is predominantly absorbed by inhalation (50%), then almost all of the Pb enters the bones (Links, Schwartz, Simon, Bandeen‐Roche, & Stewart, 2001) and can even cross the blood–brain barrier where it accumulates in the brain gray matter, especially the hippocampus (Goyer & Clarkson, 2001; Gwalteney‐Brant, 2002).

The effect of lowest level Pb exposure on the hippocampus and hippocampus‐dependent cognitive tasks was recently investigated in mouse models. In such models, low levels of exposure yielded BLL (~2 to 5 μg/dL) (Muldoon et al., 1996; Canfield, Kreher, Cornwell, & Henderson, 2003; Davis, 1990; Gilbert & Weiss, 2006; Sobin, Gutierrez, & Alterio, 2009; Nigg, Nikolas, Knottnerus, Cavanagh, & Friderici, 2010; Sobin, Parisi, Schaub, & Riva, 2011), similar to that previously measured in children (i.e., <5 µg/dl) exposed early to low Pb and showing working memory deficits (Sobin et al., 2015). Using these mice as juveniles, it was shown that lowest level Pb exposure disrupts exploratory activity (tested using the unbaited nose poke task; Flores‐Montoya & Sobin, 2015) and alters memory (tested using the novel odor recognition (NODR) task; Flores‐Montoya, Alvarez, & Sobin, 2015). Interestingly, BLLs were correlated with the extent of memory deficits in males but not in females. The functional integrity and flow of information between the hippocampus and the PFC are critical for working memory. Therefore, in an attempt to identify cellular mechanisms underlying hippocampus‐dependent behavioral deficits induced by Pb exposure, it was shown that the lowest level Pb alters microglia and decreases dentate gyrus volume in the hippocampus of Pb‐exposed mice (Sobin et al., 2013).

Pb can mimic Ca^2+^ action and/or disrupt Ca^2+^ homeostasis by entering cells through different types Ca^2+^ channels (ATSDR, 2007; Bridges & Zalups, 2005). Animal studies have also shown that different neurotransmitters and synaptic properties are affected by various levels of Pb in the hippocampus. For example, impaired dopamine neurotransmission has been observed not only in the hippocampus but also in the cerebral cortex, and cerebellum in the developing rat brain (Devi, Reddy, Prasanthi, Chetty, & Reddy, 2005; Leret, Garcia‐Uceda, & Antonio, 2002). Glutamate neurotransmission is also affected by Pb, which significantly affects long‐term potentiation (LTP; Goldstein, 1990; Gilbert & Lasley, 2007). In the hippocampus, LTP is a sustained synaptic mechanism proposed to represent the physiological substrate of learning and memory (Bliss & Collingridge, 1993; McNaughton, 1993). Hippocampal (HPC) LTP is regulated by nitric oxide, a biological messenger crucial for normal brain development and activity (Chetty et al., 2001). In fact, Pb interferes with the production of nitric oxide in many brain regions. Therefore, such effects of chronic Pb exposure on LTP (via glutamate neurotransmission and nitric oxide production) are thought to contribute to learning and memory deficits (Chetty et al., 2001). A number of laboratories have elicited LTP at glutamatergic synapses in HPC slice preparations to investigate the basis of Pb‐induced cognitive impairments (Gilbert, Mack, & Lasley, 1996, 1999; Grover & Frye, 1996; Xu et al., 1998; Zaiser & Miletic, 1997). However, aside LTP, different synaptic mechanisms and plastic properties have been described in different HPC subregions (i.e., CA1, CA2, CA3, and dentate gyrus). Such properties, crucial for shaping the overall network activity, were shown to be affected by Pb exposure. For example, short‐term synaptic plasticity elicited by paired pulse stimulation (Lasley & Gilbert, 1996; Xu et al., 1998) or long‐term depression (LTD) induction evoked by low‐frequency stimulation trains (Zhao et al., 1999) were consistently deficient in chronically exposed animals or in slices derived from them. Despite these previous scientific efforts aiming at understanding the cellular mechanisms affected by Pb in the hippocampus, the consequences of lowest level Pb exposure (leading to BLLs <10 µg/dl) on synaptic properties long after exposure has ceased, are still not well‐defined. This undermines the efforts focusing on preventive measures and therapeutic approaches.

Therefore, here, our goal was to examine the delayed consequences of early Pb exposure in HPC CA1 using a mouse model of Pb exposure. Our hypothesis was that various levels of Pb exposure would differently alter synaptic transmission and plasticity not only within CA1 but also at hippocampus–medial prefrontal cortex (mPFC) synapses which are crucial for working memory. We also anticipated that, depending on Pb levels, some synaptic changes would differ between males and females. We tested our hypothesis using field electrophysiological recordings in HPC slices from mice that had been exposed to low and high Pb levels, 8–11 months earlier, compared to nonexposed controls. We first assessed basal synaptic transmission and short‐term synaptic properties within CA1 of male and female mice. Then, we measured basal synaptic transmission and plasticity at the ventral hippocampus–mPFC synapses, using an in vitro optogenetic strategy. We propose that Pb‐induced synaptic dysfunctions might be a key therapeutic target for patients suffering from memory deficits subsequent to Pb exposure.

## 2 MATERIALS AND METHODS

### 2.1 Animals and lead exposure

A subset of the mice used in the present work come from the Flores‐Montoya et al. (2015) study, where they were used as juvenile mice. Other mice exposed to the same treatments (but not used in the Flores‐Montoya et al., 2015 study) were added to the total cohort of animals used here. All animals were used 8–11 months after the Pb exposure (as described below). All animal procedures were approved by the Institutional Animal Care and Use Committee (IACUC) of the University of Texas at El Paso (UTEP) and were carried out in accordance with the US Public Health Service Policy on Humane Care and Use of Laboratory Animals. C57BL/6J mice were purchased from Jackson Laboratories and housed in the Vivarium of the Bioscience Research Facility at UTEP. Mice were group‐housed by sex in ventilated cages with ad libitum access to food and water. Breeding was performed as previously described (Flores‐Montoya et al., 2015). Briefly, two females with one male were housed together. The females were checked daily and housed separately after vaginal plug was identified. Gestation durations ranged between 19 and 21 days. To avoid stressing dams and pups, litters remained unculled, and no other procedure was conducted during the lead exposure period (28 days). Dams typically produced litters (control = 6 litters; low‐level Pb = 4 litters; high‐level Pb = 4 litters) ranging in size from 3 to 6 pups. Each unculled litter (18 females and 24 males) was either assigned to one control treatment or one of two lead treatments as previously published (Flores‐Montoya et al., 2015; Sobin et al., 2013). To do so, pups were exposed to lead via dams’ milk from PND 0 to PND 28, where dams’ drinking water which contained either lead‐treated water (30 ppm or 330 ppm 99.4% lead acetate crystals, Sigma–Aldrich, St. Louis, MO) or sodium‐treated water (30 ppm Na^+^; control animals). Therefore, the animals were grouped into three categories: 1–0 ppm, control (*N* = 17 mice, 7 females and 10 males); 2– 30 ppm, low‐dose (*N* = 12 mice, 8 females and 4 males); and 3– 330 ppm, high‐dose (*N* = 13 mice, 3 females and 10 males). Such exposure was previously shown to result in a blood Pb level ranging between ≈2–5 μg/dL (30 ppm, low‐level exposure) and ≈10–20 μg/dL (330 ppm, high‐level exposure; see Flores‐Montoya et al., 2015; Sobin et al., 2013). These values allowed us to infer that the animals used it this study had blood Pb levels within these ranges.

### 2.2 ChR2 viral injections

We used an in vitro optogenetic approach to manipulate neurotransmitter release from HPC glutamatergic fibers that project onto mPFC neurons. To do so, unilateral viral injections were performed to specifically make excitatory HPC neurons sensitive to blue light. The rAAVDJ‐CamKIIa‐hChR2(H134R)‐eYFP (i.e., channelrhodopsin 2 [ChR2]) virus (from the Deisseroth laboratory and the Virus Vector Core of Stanford University) was injected in anesthetized mice placed in a stereotactic apparatus. A viral construct devoid the opsin gene (pAAVDJ‐CamKIIa‐eYFP; from the Deisseroth laboratory and the Virus Vector Core of Stanford University) was used in control experiments, in separate animal cohorts. In all cases, 200 nl of the viral particles were injected in the hippocampus at a flow rate of 100 nl/min through a glass micropipette. The injection sites (relative to Bregma) were the following: (a) −3.05 mm anterior/posterior (AP), 2.65 mm medial/lateral (ML), and −1.40 mm and −4.55 mm dorsal/ventral (DV); (b) −3.05 mm AP, 3.00 mm ML, and −1.71 mm and −4.25 mm DV; (c) −3.05 mm AP, 3.35 mm ML, and −2.30 mm and −3.90 mm DV; (d) −3.05 mm AP, 3.70 mm ML, −2.92 mm, and −3.17 mm and −3.30 mm DV. The animals were allowed to recover for 4–6 weeks after which electrophysiological recordings were obtained.

### 2.3 Electrophysiology in hippocampal and medial prefrontal cortex slice preparations

Experiments were performed on 9‐ to 12‐month‐old mice. Isoflurane was used to anesthetize mice that were then decapitated. After a skull incision, the brain was removed and placed in ice‐cold dissecting solution (in mM): 195 sucrose, 10 NaCl, 2.5 KCl, 1 NaH_2_PO_4_, 25 NaHCO_3_, 10 glucose, 4 MgSO_4_, and 0.5 CaCl_2_. Transverse (HPC for in vitro electrophysiological experiments) or coronal (HPC and mPFC for in vitro electrophysiological/optogenetics experiments) brain sections were cut using a vibratome (Leica VT1200S). The freshly cut brain slices were immediately transferred to an interface chamber and allowed to recover for at least 2 hr at 34°C–36°C. Once placed in the interface chamber, the slices were continuously perfused with artificial cerebrospinal fluid (aCSF) (bubbled with 5% CO2/95% O2), which had the following composition (in mM): 124 NaCl, 2.5 KCl, 1 NaH_2_PO_4_, 25 NaHCO_3_, 10 glucose, 1 MgSO_4_, and 2 CaCl_2_. The aCSF was maintained at 34–36°C and fed by gravity at a rate of 2–3 ml/min. Field electrophysiological recordings were made via a glass microelectrode (3–5 MΩ). This recording microelectrode was made of pulled borosilicate glass capillary, which was then filled with aCSF. The recording microelectrode was placed in the CA1 stratum radiatum of HPC slices or placed in layer 5 (L5) of mPFC slices (∼550 µm from midline, within the mPFC infralimbic area). In HPC slices, the electrical stimulating electrode used to activate the Schaffer collateral (SC, from CA3 neurons) was typically placed 200 µm away from the CA1 HPC recording site. The electric stimulations were delivered to the SC using stimulus intensities ranging between 0.1 and 2.1 mV. The in vitro optogenetic/extracellular field electrophysiological recordings were carried out in mPFC slices, ipsilateral to the viral injection site. An optical fiber coupled to a blue LED module (473 nm, Plexon) was used to photostimulate the ChR2‐expressing neuronal fibers in mPFC slices. The optical fiber mounted on a micromanipulator was usually placed about 50–100 µm away from the L5 recording site. The light‐evoked field synaptic responses recorded in the mPFC slices resulted from the photostimulation of HPC ChR2‐expressing afferent fibers. Field excitatory postsynaptic potentials (fEPSPs) consisted of a small, initial, non‐synaptic fiber volley (whose amplitude is proportional to the number of recruited afferent fibers), followed by a prominent excitatory postsynaptic response.

To determine the effect of Pb exposure on neuronal activity, basal synaptic transmission was first assessed. Basal synaptic transmission was recorded at 0.033 Hz, at increasing stimulation intensities, yielding fEPSPs of increasing amplitude. We used either electrical stimulations (electrophysiological experiments) or photostimulations (optogenetic/extracellular field electrophysiological experiments). The electrical stimulation pulse duration was 0.1 ms, whereas the photostimulating pulse duration (where 1 r. l. u. of light intensity = 0.43 mW) was 1 s. To determine the effect of Pb on neuronal fiber excitability, the amplitude of the fiber volley was also evaluated. To perform the subsequent synaptic plasticity assays (i.e., paired stimulations and short stimulation trains), the stimulus intensity that generated a fEPSP one third of the maximum synaptic response obtained during the basal synaptic transmission assay was selected. Therefore, using the selected stimulation intensity, we then determined whether Pb acted at presynaptic sites by using two short‐term plasticity assays, thought to involve presynaptic mechanisms: (a) to determine whether Pb exposure affects the presynaptic initial probability of neurotransmitter release, short‐term synaptic plasticity was induced using paired stimulations (electrical or light‐induced) 20, 50, 100, 200, 400, and 800 ms apart and (b) to assess whether Pb exposure affects presynaptic neurotransmission at physiological frequency ranges, short‐term synaptic depression assays were used. To do so, 7‐pulse electrical stimulation trains were applied at 20 and 50 Hz (electrophysiological experiments) or 40‐pulse photostimulation trains were applied at 5, 10, and 20 Hz (optogenetic/electrophysiological experiments). For all groups, fEPSPs recorded during the short‐term depression (STD) experiments were normalized to the fEPSP value elicited by the first stimulation pulse. Finally, the effect of Pb exposure was determined at hippocampus‐to‐mPFC synapses, known to be crucial for working memory, by assessing the basal synaptic transmission and short‐term plasticity assays described above. The glutamatergic receptor blockers CNQX (25 µM, Ascent Scientific) and AP5 (50 µM, TOCRIS) were bath applied during recordings to confirm the chemical identity of the excitatory synapses investigated. Fiber volley was quantified by measuring the amplitude of the first peak negativity of the field responses, and the field postsynaptic responses were quantified by measuring the initial slope of the second peak negativity of the responses. The electrophysiological signals were acquired using the pClamp10 software (Molecular Devices), the Digidata 1440A (Molecular Devices), and an extracellular amplifier (Cygnus Technologies).

### 2.4 Imaging/fluorescence analysis

To evaluate and compare the expression of ChR2‐eYFP between brain slices, a subset of vHPC and mPFC slices were fixed with 4% paraformaldehyde in phosphate buffer saline (PBS), following the in vitro optogenetic/electrophysiological recordings. Tissue slices were then washed with TBS (5 washes, 5 minutes each) and incubated in blocking solution (2% normal donkey serum, 0.1% Triton X‐100; in TBS) for 1–2 hr at room temperature. Tissue sections were then incubated in primary antibodies (GFP at 1:1,000 dilution, Millipore; GAD67 at 1:200 dilution, ThermoFisher Scientific) for 60 hr at 4°C. Tissue was then washed and incubated in respective secondary antibody (Alexa Fluor 488 and Cy3 at 1:500 dilution, Jackson ImmunoResearch Laboratories) for 4–5 hr at room temperature. Tissue sections were mounted on glass slides and coverslipped. Slides were scanned on a LSM 700 Confocal microscope with a 20× objective using the ZEN software. Background for each brain slice was subtracted by using a tissue area devoid of GFP staining. The gain was adjusted to avoid saturation levels at both the injection site (with a value of 504 at the vHPC) and the projection site (with a value of 682 at the mPFC). The regions of interest analyzed for fluorescence was encircled with an average standard deviation of 16.9 for vHPC slices and of 13.03 for mPFC slices. The fluorescence intensity values of the regions of interest were acquired and analyzed using the ZEN software. Image analysis was conducted blind to group type. A total of 24 images from 6 mice were analyzed.

### 2.5 Statistical analyses

Statistical analyses were performed using the *R* statistical software package (R Core Team, 2015), linear mixed effect package *lme4* (Bates, Mächler, Bolker, & Walker, 2015), *lsmeans* package (Lenth, 2016), and the *multcomp* package (Hothorn, Bretz, & Westfall, 2008). Polynomial mixed effects models were used to assess the change in synaptic transmissions for each group across time. This is an effective method to model data where measures (stimulus levels) are nested within subjects (mice) and may induce complex correlation structures. The responses analyzed using this strategy include: (a) the extracellular fEPSPs, (b) the fiber volley amplitude, (c) the paired‐pulse ratio (PPR), and (d) STD elicited by short stimulation trains at different frequencies. In each model, the random effect was the mouse. The mouse random effects allow for modeling of the individual mouse trajectories in a single linear model and describe deviations of an individual mouse from the group average. Up to cubic degree polynomial terms were used to analyze the relationship between synaptic transmissions and the stimulus level. The polynomial terms for stimulus levels were allowed to interact with the lead exposure group and, separately, the sex of the mouse. Power analysis indicates that with the parameterization utilized for each dependent variable modeling, we can be assured reasonable power levels for each mixed model (see Table 1) when assuming a 5% significance level. The fixed effect parameters of the model summarize the population averaged effects and are useful for overall trends. The least squares means presented are based on these models, and use efficient and robust standard errors resulting from the linear mixed model. Post hoc *t* tests on the log scale were used to compare differences between groups. Since all lead group comparisons within sex and stimulus level were of interest, all intervals are adjusted for multiplicity using the Tukey's correction. Data are presented as means ± *SEM*. “N” indicates the number of animals, “n” indicates number of slices, and “*df*” indicates the error degree of freedom group comparison associated with the mixed model. The “*df*” may be fractional due to use of the Satterthwaite approximation. All recordings and the majority of data analyses were done blind to the Pb group or sex.

**Table 1:** Statistical parameters and values

Dependent variable	Num *df*, Denom *df*	Effect size	Estimated power
Io.f	61, 975	0.05	96.6%
50 Hz	154, 161	0.25	83.7%
20 Hz	142, 154	0.25	82.7%
Ppr	204, 252	0.25	96.0%

## 3 RESULTS

### 3.1 Early Pb exposure alters CA3–CA1 hippocampal synaptic transmission in adult mice

**Figure 1:**
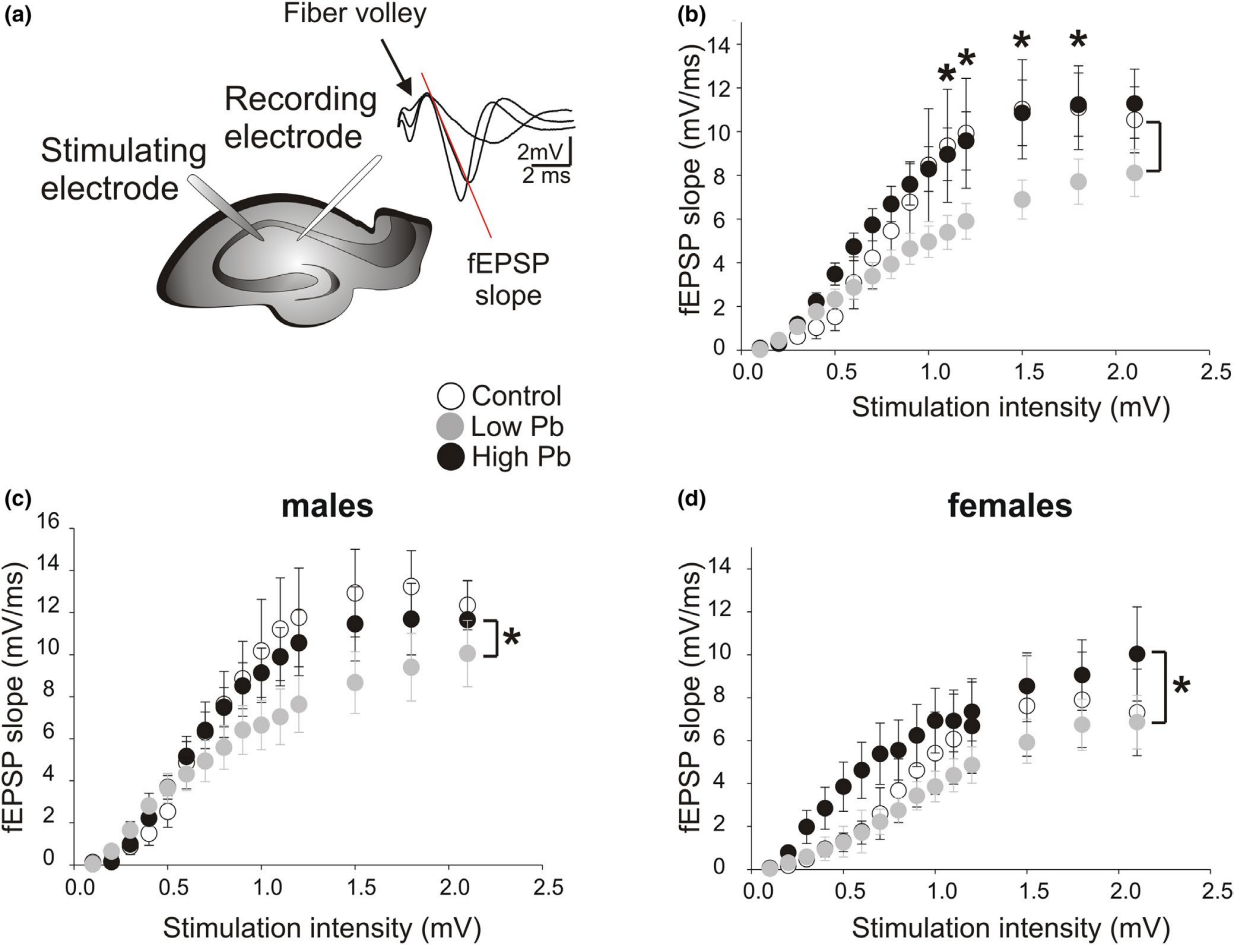
The consequence of early Pb exposure on synaptic transmission of CA1 hippocampal neurons in adult mice. (a) *Left*, Schematic representation of a transverse hippocampal slice including the CA3 and CA1 areas. The electrical stimulating electrode was placed on CA3 Schaffer collateral fibers. The extracellular recording electrode was placed in the stratum radiatum of CA1. *Right*, Sample traces recorded in response to increasing stimulation intensities and showing the fiber volley (arrow) as well as the fEPSP initial slope (red line). (b) Plot showing the mean field excitatory postsynaptic potentials (fEPSPs) initial slopes as a function of stimulation intensity (i.e., stimulus–response curve) for all mice tested under the three following experimental conditions: 1—control, 2—high Pb exposure, and 3—low Pb exposure. (c) Plot showing the stimulus–response curve for the male mice under control conditions, high Pb exposure, and low Pb exposure. (d) Plot showing the stimulus–response curve for the female mice under control conditions, high Pb exposure, and low Pb exposure. In (b)–(d): Control = open circles; High Pb exposure = black circles; Low Pb exposure = gray circles. Individual circles represent the mean value (±SE) obtained at each stimulation intensity. *p < 0.05

Hippocampus‐dependent cellular and behavioral impairments were previously observed in preadolescent C57BL/6J mice exposed early to high and low Pb levels (Flores‐Montoya et al., 2015; Sobin et al., 2013). Therefore, using a subset of these mice along with a group of mice exposed to lowest and high Pb levels, we performed in vitro extracellular field recordings in HPC slices. This was done to characterize the long‐term consequences of early Pb exposure on synaptic properties in CA1, 8–12 months postexposure. Electrical stimulation of CA3 fibers were used to evoke fEPSPs in the CA1 stratum radiatum (Figure 1a). A stimulus–response curve was first plotted to quantify and compare the CA3–CA1 basal synaptic transmission in adult mice that were exposed early (i.e., from PND 0 to PND 28) to either low or high Pb levels. Figure 1b shows that at the lowest stimulation intensities (0.1–1 V), there was no difference between groups. However, at higher stimulation intensities (1.1–1.8 V), low Pb exposure was associated with a smaller synaptic strength. At these intensities, the fEPSP slopes recorded in the low Pb‐exposed mice (*N* = 12 animals, *n* = 19 slices, *df* = 91) were lower than that of the nonexposed, control mice (*N* = 17 animals, *n* = 24 slices, *df* = 91; *p = *0.0065). Control and high Pb‐exposed (*N* = 13 animals, *n* = 22 slices, *df* = 91) animal were not different (*p* > 0.05*)*.

Interestingly, early Pb exposure had different effects when males and females were analyzed separately (Figure 1c,d, respectively). That is in males (Control: *N* = 10, *n* = 16, *df* = 25; High Pb: *N* = 10, *n* = 16, *df* = 25; Low Pb: *N* = 4, *n* = 8, *df* = 25), synaptic transmission was lower in low Pb‐exposed mice compared to control mice (*p = *0.0017). Synaptic transmission of the control versus the early high Pb‐exposed male mice was not different (*p* = 0.3451). In contrast in females (Control: *N* = 7, *n* = 8, *df* = 25; High Pb: *N* = 3, *n* = 6, *df* = 25; Low Pb: *N* = 8, *n* = 11, *df* = 25), the synaptic transmission of the early low Pb‐exposed female mice versus the control mice was not different (*p = *0.9960). However, synaptic transmission (0.1–1.1 V) was higher in female mice exposed early to high Pb compared to control mice (*p = *0.0069). In summary, our data suggest that early lowest Pb exposure significantly decreases synaptic transmission in adult males, whereas early high Pb exposure significantly increases synaptic transmission in adult females.

### 3.2 Early Pb exposure modifies CA3 axonal excitability in the hippocampus of adult mice

**Figure 2:**
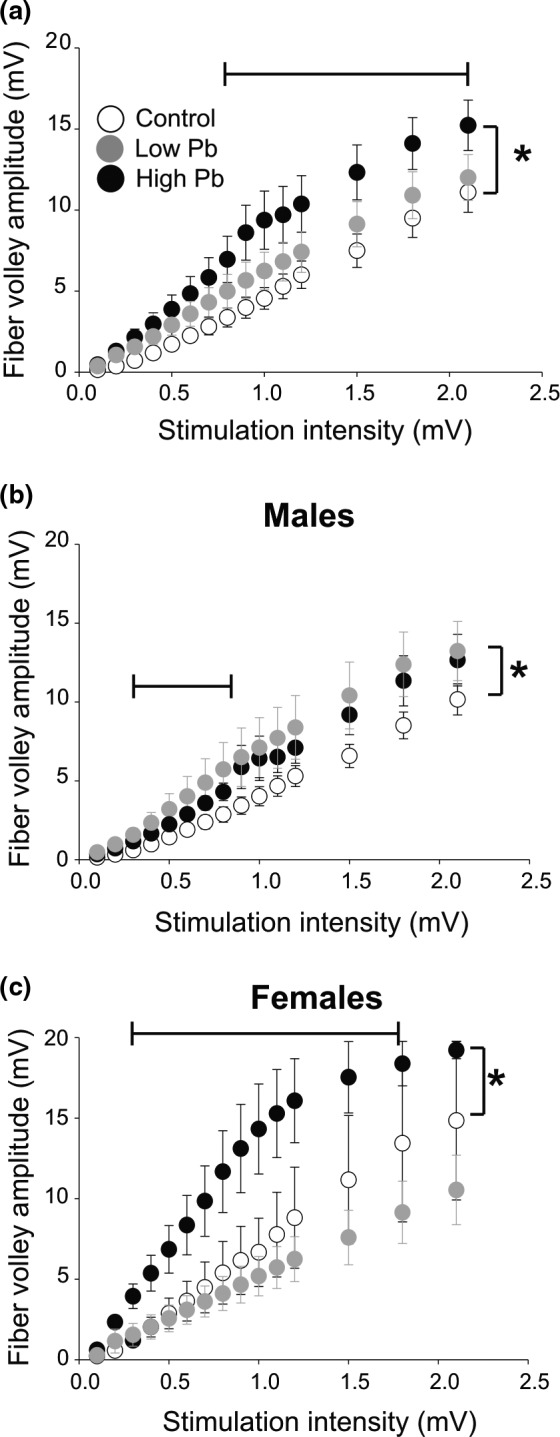
The consequence of early Pb exposure on the excitability of the CA3 afferent fibers in adult mice. (a) Plot of CA3 fiber volley amplitudes (recorded in CA1) as a function of stimulation intensity. Data plotted for all the animal tested under the three experimental conditions: 1—control, 2—high Pb exposure, and 3—low Pb exposure. (b) Plot showing the fiber volley amplitude for the male mice under control conditions, high Pb exposure, and low Pb exposure. (c) Plot showing the fiber volley amplitude for the female mice under control conditions, high Pb exposure, and low Pb exposure. In (a)–(c): Control = open circles; High Pb exposure = black circles; Low Pb exposure = gray circles. Individual circles represent the mean value (±SE) obtained at each stimulation intensity. *p < 0.05

The amplitude of evoked synaptic responses depends on the number of synapses, the release probability and the vesicular content (i.e., quantal size) of presynaptic boutons. Therefore, each of these variables could contribute to the above Pb‐dependent change in synaptic strength. Thus, next, we measured the amplitude of the non‐synaptic fiber volley at CA3–CA1 synapses, as this small presynaptic response is proportional to the number of activated axons associated with functional synapses. The data in Figure 2a show that early high‐level Pb exposure is associated with a significant increase in fiber volley amplitude (*N* = 9, *n* = 10; *p* < 0.0001), compared to control conditions (*N* = 9, *n* = 10). Early low‐level Pb exposure (*N* = 7, *n* = 11) did not alter fiber volley amplitude (*p* > 0.05).

Similar to the basal synaptic transmission data, Pb exposure produced different effects on fiber volley amplitude when males and females were analyzed separately (Figure 2b,c, respectively). Based on the basal synaptic transmission data described above for male mice, we hypothesized that early low Pb exposure would be associated with a smaller fiber volley amplitude (see Figure 1c). However, in males (Figure 2b), contrary to our expectations, the fiber volley amplitude of the low Pb‐exposed males was greater (*N* = 3, *n* = 6; *p = *0.0095) than that of the control mice (*N* = 7, *n* = 8). The fiber volley amplitude of the high Pb‐exposed mice (*N* = 6, *n* = 7) versus controls was not different (*p* = 0.5289). In females (Figure 2c), as expected, the fiber volley amplitude of the control (*N* = 2, *n* = 2) versus the early low Pb‐exposed females (*N* = 4, *n* = 5) was not different (*p* > 0.05). The fiber volley amplitude of the high Pb‐exposed mice (*N* = 3, *n* = 3) increased by twofold increase compared to control mice (*p* < 0.0001). Together, these data suggest that in adult male mice, early low Pb exposure increases the number of activated neuronal fibers and decreases synaptic transmission. In adult female mice, early high Pb exposure increases the number of activated neuronal fibers and increases basal synaptic transmission.

### 3.3 Neurotransmitter release probability in adult mice is not modified by early Pb exposure

**Figure 3:**
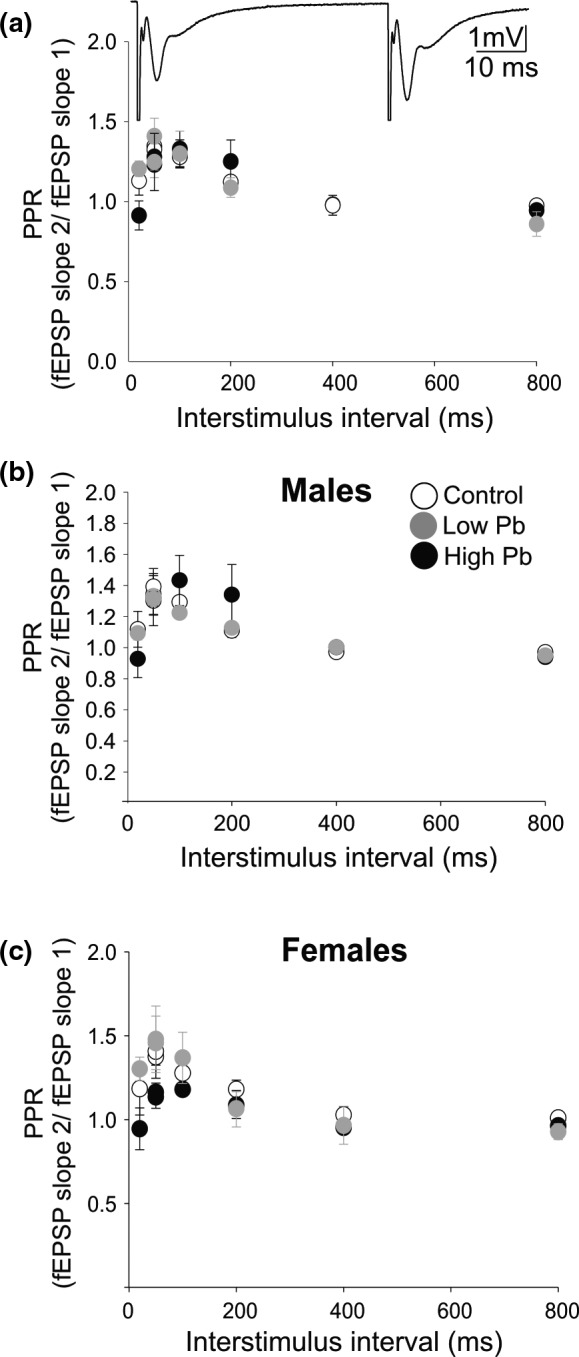
Early Pb exposure did not affect short‐term synaptic facilitation in the CA1 region of the hippocampus of adult mice. (a) Plot showing the paired‐pulse ratio (PPR) recorded at CA3–CA1 synapses for all animals in control and exposed to Pb. The pulses were applied at interstimulus intervals (ISIs) of: 50, 100, 200, 400, and 800 ms under the three experimental conditions: 1—control, 2—high Pb exposure, and 3—low Pb exposure. A paired‐pulse sample trace is shown at the top with an ISI of 50 ms. (b) Plot showing the PPR for the male mice under control, high Pb exposure, and low Pb exposure experimental conditions. (c) Plot showing the PPR for the female mice under control, high Pb exposure, and low Pb exposure. In (a)–(c): Control = open circles; High Pb exposure = black circles; Low Pb exposure = gray circles. Individual circles represent the mean value (±*SE*) obtained at each interstimulus interval (ISI)

Next, the initial probability of neurotransmitter release was assessed to determine if a Pb‐induced change in neurotransmitter release could be associated with the changes in basal synaptic transmission shown above in Figure 1. To assess the fractional neurotransmitter release at CA3–CA1 synapses, we used a paired‐pulse protocol at various interstimulus intervals (ISIs) (Figure 3). Changes in paired‐pulse ratio (PPR) generally reflect changes in presynaptic neurotransmitter release efficacy (Xu‐Friedman & Regehr, 2004). At ISIs shorter than 400 ms, facilitation of the fEPSPs was observed in all mice, as previously described (Drew et al., 2011; Fénelon et al., 2011), suggesting that, in both control and Pb‐exposed mice, the synapses between the Schaeffer collaterals and the CA1 neurons have a low initial probability of neurotransmitter release (Figure 3a). Such short‐term synaptic plasticity was absent at ISIs greater than 400 ms. Because PPR was unchanged between the animal groups (Wild Type [WT]: *N* = 10, *n* = 10, *df* = 204; High Pb: *N* = 13, *n* = 17, *df* = 204; Low Pb: *N* = 10, *n* = 16, *df* = 204) (low t = −0.252, *p = *0.9655; high t = −0.564, *p* = 0.8392), our data suggest that the probability of neurotransmitter release is unaffected by early Pb exposure. Furthermore, this result was seen in both males (WT: *N* = 7, *n* = 7, *df* = 204; High Pb: *N* = 8, *n* = 10, *df* = 204; Low Pb: *N* = 4, *n* = 7, *df* = 204) (low *t* = 0.191, *p* = 0.8077; high *t *= −0.416, *p* = 0.9089) and females (WT: *N* = 3, *n* = 3, *df* = 204; High Pb: *N* = 5, *n* = 7, *df* = 204; Low Pb: *N* = 6, *n* = 9, *df* = 204) (low *t *= −0.103, *p* = 0.9942; high *t* = 1.460, *p* = 0.3123) (Figure 3b,c).

### 3.4 Hippocampal short‐term synaptic depression is unchanged in adult mice exposed to Pb early in life

**Figure 4:**
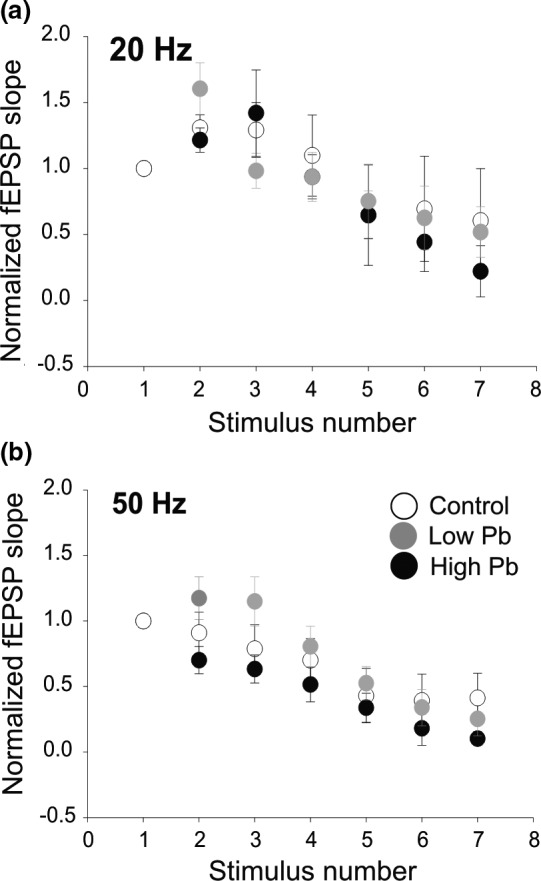
Short‐term synaptic depression is unaltered by early Pb exposure in the CA1 region of the hippocampus of adult mice. (a) Plot showing an initial facilitation followed by a short‐term depression (STD) recorded at CA3–CA1 synapses at 20 Hz. STD is similar between control, high Pb‐exposed, and low Pb‐exposed mice. (b) Plot showing the STD recorded at CA3–CA1 synapses at 50 Hz. STD is similar among all groups. In (a) and (b): Control = open circles; High Pb exposure = black circles; Low Pb exposure = gray circles. Individual circles represent the mean value (±*SE*) obtained at each interstimulus interval (ISI)

During normal hippocampus‐dependent memory tasks, CA1 neurons can receive trains of inputs from neighboring cells in the 20–60 Hz frequency range (reviewed by Igarashi, 2015). Therefore, next, we wanted to determine if early Pb exposure could affect synaptic transmission within this physiological frequency range. To mimic physiological synaptic inputs, we electrically activated the CA3–CA1 synapses using short trains of 7 pulses at 20 and 50 Hz (Figure 4a,b).

When the three animal groups were compared at 20 Hz, the 7‐pulse stimulation train produced a 50% STD of the fEPSPs (Figure 4a) which did not differ between groups (WT: *N* = 5, *n* = 5, *df* = 154.04; High Pb: *N* = 5, *n* = 7, *df* = 154.04; Low Pb: *N* = 7, *n* = 11, *df* = 154.04, low t=−0.702, *p = *0.7628). Similarly, at 50 Hz, the fEPSPs showed a 50%–75% STD (Figure 4b). At this frequency, the shape of the STD elicited by the train of 7 pulses did not differ between the groups (low *t* = −1.999, *p = *0.1,158) (high *t* = 0.432, *p* = 0.9022). These results suggest that early Pb exposure does not affect STD at CA3–CA1 synapses, when assessed more than 8 months after Pb exposure has ceased.

### 3.5 Synaptic transmission at vHPC–mPFC synapses is reduced in adult mice exposed early in life to lowest level Pb

**Figure 5:**
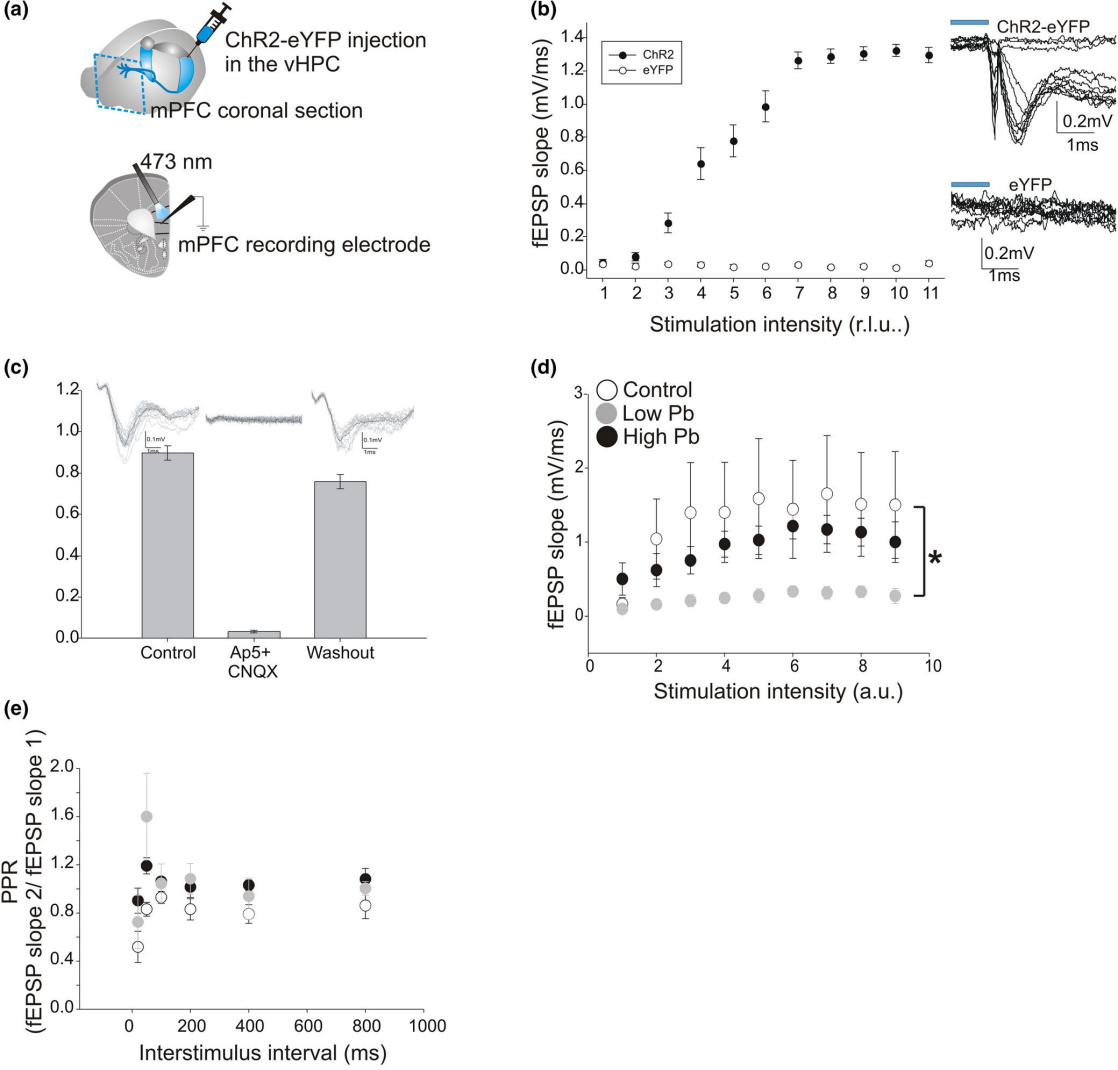
Synaptic transmission and facilitation at the ventral hippocampus‐medial prefrontal cortex (vHPC–mPFC) synapses of Pb‐exposed adult mice. (a) Schematic illustration of the unilateral injection site in the hippocampus. The 473 nm light and the recording electrode placement in the mPFC coronal slice are identified and field excitatory postsynaptic potentials (fEPSPs) examples traces are shown at photostimulations of increasing intensity. (b) Plot showing that the light‐evoked fEPSPs are due to the expression of channelrhodopsin 2 (ChR2). In mice injected with eYFP only (i.e., lacking ChR2), photostimulations failed to elicit fEPSPs. (c) Plot of light‐evoked fEPSPs before (control), during, and after (washout) treatment with CNQX and APV. *Insert*, sample traces of light‐evoked fEPSPs responses recorded in mPFC under these experimental conditions. (d) Plot summarizing the optically evoked fEPSPs at vHPC–mPFC synapses in control, high Pb‐exposed, and low Pb‐exposed mice. Low Pb exposure significantly reduced synaptic transmission at vHPC–mPFC synapses. (e) Paired‐pulse ratios (fEPSP slope 2/fEPSP slope 1) of vHPC–mPFC synapses are not affected by Pb exposure at all the interstimulus intervals tested. *p < 0.05

Previous work in rodents has shown that the CA1 and subicular subdivisions of the ventral hippocampus send ipsilateral, unidirectional, and glutamatergic projections to the mPFC (Jay, Thierry, Wiklund, & Glowinski, 1992; Jay, Glowinski, & Thierry, 1995). Moreover, previous human studies showed that early low Pb exposure yielding BLL of <5 µg/dl causes working memory deficits in children (Canfield et al., 2003; Sobin et al., 2015). From our above CA1–CA3 results, we hypothesized that early Pb exposure could alter the function of vHPC–mPFC synapses in adult mice. To test this hypothesis, ChR2 was injected in the mouse vHPC to make CA1 neurons sensitive to blue light (Figure 5a). Photostimulation of the ChR2‐expressing afferent fibers (originating from the vHPC) elicited glutamate receptor‐dependent fEPSPs in L5 of acute mPFC slices, which were abolished in the presence of AP‐5 and CNQX (Figure 5a–d). A stimulus–response analysis revealed that at all the photostimulation intensities applied, the light‐evoked fEPSPs were significantly smaller in the low Pb‐exposed mice (*N* = 2, *n* = 3, *df* = 59) compared to nonexposed controls (*N* = 5, *n* = 6, *df* = 59. low vs. con *t* = −2.801, *p* = 0.0111) or compared to the high Pb‐exposed mice (*N* = 4, *n* = 8, *df* = 59) (*p < *0.05*)*.

Then, paired‐pulse stimulation was performed at vHPC–mPFC synapses to determine whether the differences in synaptic strength between these animal groups were due to differences in the probability of neurotransmitter release (Figure 5e). Paired‐pulse evoked by photostimulation revealed normal short‐term synaptic transmission in both the groups of Pb‐exposed mice at various interpulse intervals (low *t* = −1.074, *p* = 0.4880; high *t* = 0.089, *p* = 0.9903). These data suggest that the fractional vesicular release at vHPC–mPFC synapses is unaffected with time, by early Pb exposure.

### 3.6 Short‐term depression is altered at vHPC–mPFC synapses of adult mice exposed early in life to high‐level Pb

During spatial working memory tasks, neural activity in the mPFC becomes synchronized with the HPC theta oscillations, that is, ≈4–12 Hz. Therefore, we then investigated if early Pb exposure could alter vHPC–mPFC synaptic strength using stimulation frequencies relevant to spatial working memory in adult mice. To test our hypothesis in mPFC slices, we photostimulated ChR2‐expressing HPC inputs at frequencies not only within the theta range (i.e., at 5 and 10 Hz) but also outside the theta range (20 Hz), as a control.

**Figure 6:**
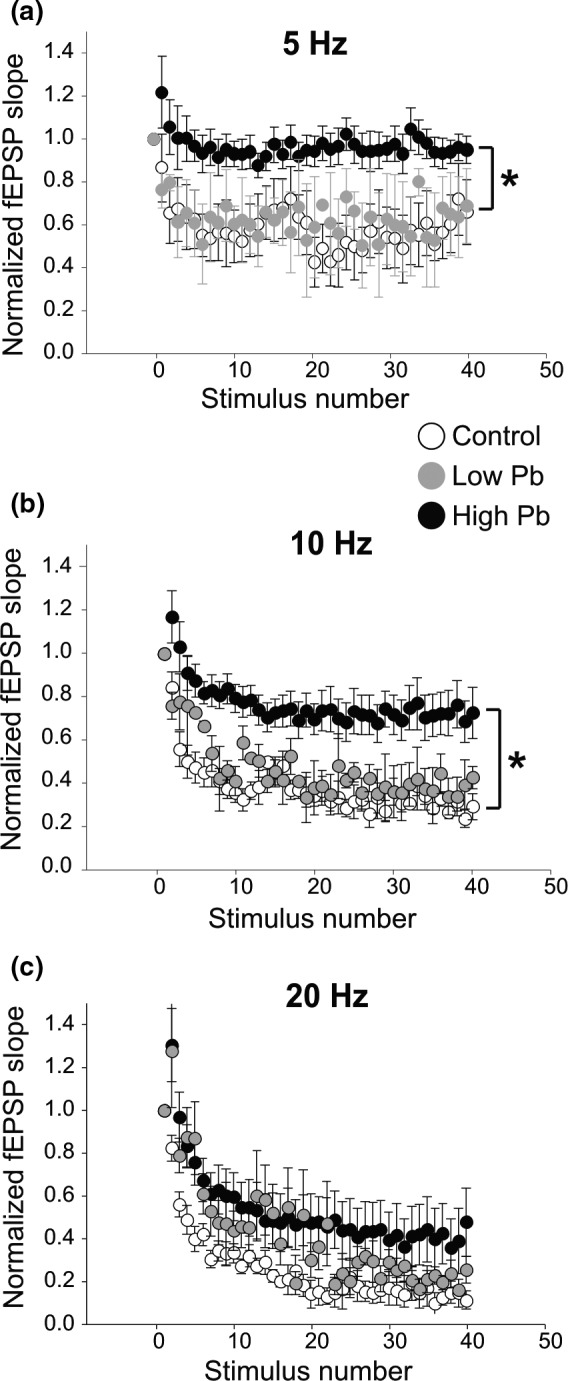
Early Pb exposure alters short‐term synaptic depression at the ventral hippocampus‐medial prefrontal cortex (vHPC–mPFC) synapses of adult mice. (a) Plot showing an initial facilitation followed by a short‐term depression (STD) recorded at the vHPC–mPFC synapses at 5 Hz. STD was significantly decreased in high Pb‐exposed mice compared to control and low Pb‐exposed mice. (b) Plot showing an initial facilitation followed by STD recorded at the vHPC–mPFC synapses at 10 Hz. STD was significantly lower in high Pb‐exposed mice compared to control and low Pb‐exposed mice. (c) Plot showing an initial facilitation followed by STD recorded at the vHPC–mPFC synapses at 20 Hz. At that frequency, STD was not different between the different animal groups. In (a)–(c): Control = black circles; High Pb exposure = grey circles; Low Pb exposure = white circles. Individual circles represent the mean value (±SE). *p < 0.05

As can be seen in Figure 6a, in both control and low Pb‐exposed animals, the 5 Hz photostimulation train produced a ≈35%–40% STD of the fEPSPs (normalized fEPSP slopes evoked by the last 10 photostimulations: Control, 0.60 ± 0.04 and low Pb, 0.65 ± 0.05) that did not differ between these two animal groups (Control: *N* = 5, *n* = 6, *df* = 154.04. Low Pb: *N* = 2, *n* = 3, *df* = 154.04; *t* = 0.228, *p* = 0.9527). However, the STD of the high Pb‐exposed mice (*N* = 4, *n* = 8, *df* = 154.04) was different than the STD of the other two animal groups (high *t* = 2.772, *p* = 0.0284). That is, in high Pb‐exposed mice, the second and third fEPSPs were facilitated and the subsequent fEPSPs showed a very modest ≈3% STD (Normalized fEPSP slopes evoked by the last 10 photostimulations: High Pb, 0.97 ± 0.02). This STD was significantly smaller than that of the control and low Pb‐exposed animals.

The same effects of early high Pb exposure on STD were observed at 10 Hz (Figure 6b). High Pb exposure led to a ≈30% STD (normalized fEPSP slopes of the last 10 photostimulations: High Pb: 0.73 ± 0.03). This STD was significantly smaller than that of the ≈ 70% STD obtained from the control (high *t* = −4.255, *p = *0.0016) and low Pb (high vs. low *t* = 7.356, *p < 0.0001*) exposed animals. The STD of control versus low Pb‐exposed animals was not significantly different (normalized fEPSP slopes of the last 10 photostimulations: Control: 0.31 ± 0.01 and low Pb: 0.38 ± 0.03) (low *t* = 0.6924, *p = *0.7124).

Interestingly, the effect of early Pb exposure on the activity of vHPC–mPFC synapses stimulated at frequencies outside the theta range (i.e., 20 Hz, Figure 6c) did not yield significant difference among the three animal groups (low vs. con *t* = 1.01, *p = *0.5198; high *t* = 2.186, *p* = 0.0877). In fact, at 20 Hz, the three groups showed a 70%–85% decrease in fEPSP slope (normalized fEPSP slopes of the last 10 photostimulations: Control: 0.15 ± 0.02; High Pb: 0.41 ± 0.04; Low Pb: 0.23 ± 0.02). These data suggest that early high Pb exposure affects the STD of vHPC–mPFC synapses at frequencies only within the theta range relevant to spatial working memory in adult mice.

**Figure 7:**
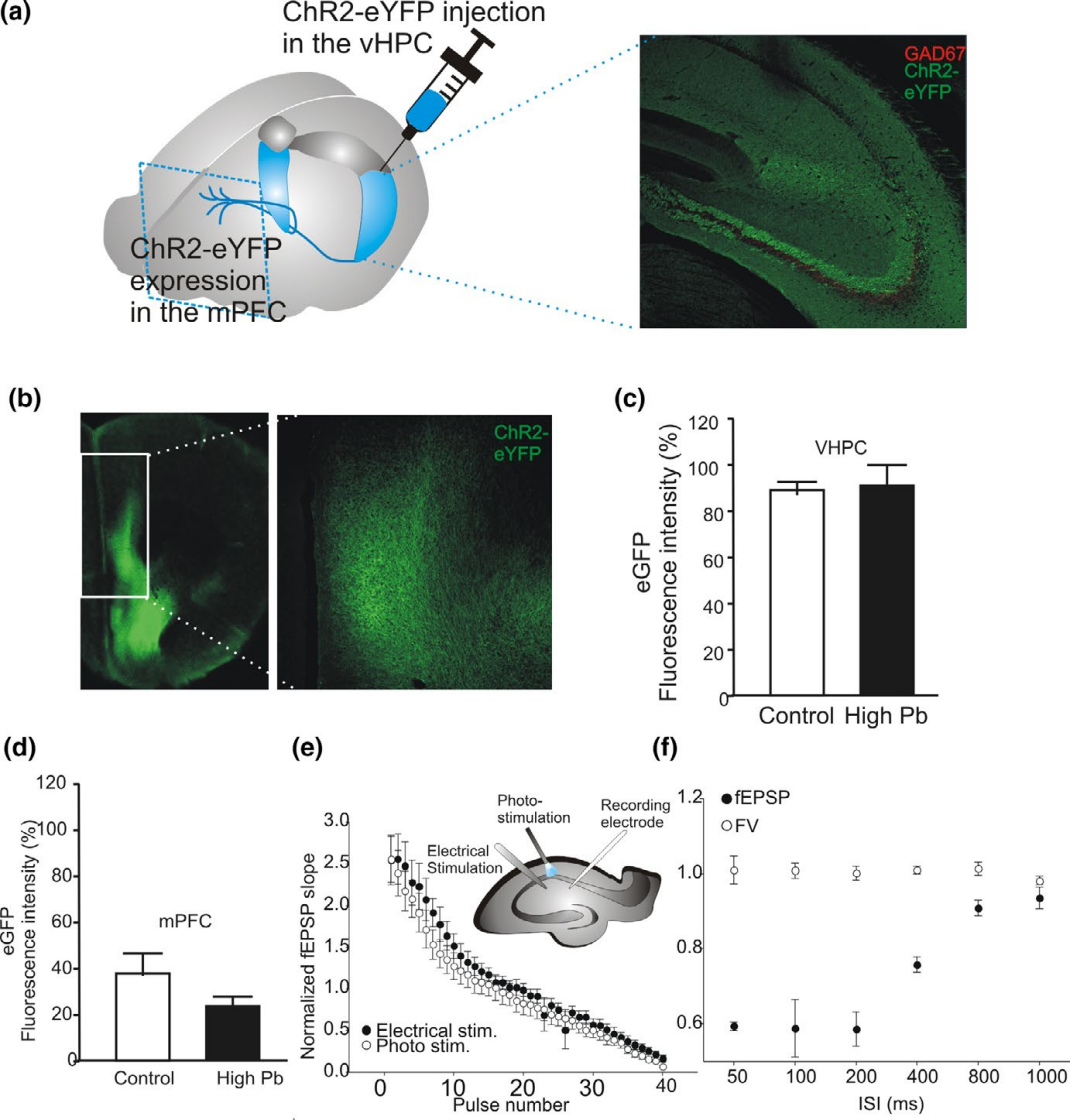
Analysis of ChR2‐eYFP expression in the sites of viral injection (ventral hippocampus [vHPC]) and projection (medial prefrontal cortex [mPFC]). (a) Confocal image of the ipsilateral vHPC showing eYFP‐labeled cell bodies (green) that are not GAD67 immunopositive (red) in the stratum pyramidal. (b) mPFC tissue section ipsilateral to the injection site showing dense eYFP‐labeled fibers (green). (c) Quantification of the intensity of eYFP fluorescence in the stratum oriens/pyramidale of the CA1 area in control mice was not different from that of mice exposed to Pb. (d) Quantification of the intensity of eYFP fluorescence in layer 5 of the mPFC in control mice was similar to that of mice exposed to Pb. (e) Plot showing the short‐term depression (STD) recorded at CA3–CA1 synapses at 20 Hz with electrical and photostimulation. STD is similar between the two methods of stimulation. (f) Paired‐pulse ratios (fEPSP slope 2/fEPSP slope 1) at the vHPC shows presynaptic depression, while the fiber volley (FV) is not significantly changed among interstimulus intervals (ISIs)

The possibility that alterations in ChR2 transduction/expression or function account for differences in synaptic response magnitude is important to consider. To address this, following control experiments were performed to confirm that: (a) ChR2‐eYFP expression was restricted to excitatory cell bodies in the stratum pyramidal of the HPC; (b) ChR2‐eYFP expression of virally infected brain regions was similar between animals; and (c) optically induced activity was as expected and comparable to electrical stimulation in HPC slices (Figure 7). Regarding ChR2‐eYFP expression at the HPC injection site, eYFP‐labeled cell bodies were not colabeled with the GABAergic marker, GAD67 (Figure 7a; *N* = 4, *n* = 16). Moreover, such eYFP‐labeled neuronal fibers were present in the ipsilateral mPFC (Figure 7b). Fluorescence intensity analysis also confirmed equivalent ChR2‐eYFP expression in vHPC and mPFC slices of Pb‐exposed and control mice (Figure 7c,d; *N* = 2 control mice; *N* = 3 Pb‐exposed mice; *t* test, *p* > 0.05). These results therefore confirmed that our photostimulation protocols activated a comparable number of vHPC–mPFC excitatory synapses between the different animal groups. Finally, we compared the outcome of the two stimulation methods to ensure that the photoactivation of ChR2‐expressing neuronal fibers mimicked their electrical activation. Our results show no significant difference between fEPSPs recorded at CA3–CA1 synapses and evoked at 20 Hz using either an electrical stimulation or a photostimulation (Figure 7e; *N* = 4; *n* = 8). Similarly, a photostimulation‐induced STD of the fEPSPs was recorded at CA3–CA1 synapses, without affecting the fiber volley amplitude (Figure 7f; *N* = 4; *n* = 8). This suggests that the photostimulation induces synaptic plasticity and reliably recruits fibers, mimicking an electrical stimulation.

Overall, these data are consistent with the hypothesis that early Pb exposure disrupts the efficacy of vHPC inputs to the mPFC. Such Pb‐induced disruption may therefore contribute to long‐term deficits in working memory as seen in children exposed to Pb early in life.

## 4 DISCUSSION

Currently, more studies are investigating the effects of chronic low‐level Pb exposure on clinically relevant cognitive processes and brain neurochemistry. However, few studies examined the sex‐specific consequences of early chronic Pb exposure on neuronal function, long after Pb exposure has ceased. Such knowledge would promote preventive measures against Pb‐induced neurotoxic effects. Here, using mice, we characterized the neuronal consequences of early chronic Pb exposure, following a prolonged postexposure period. We show complex effects of Pb exposure on the activity of HPC neurons as assessed by our in vitro electrophysiological recordings, 8–11 months postexposure. More precisely, Pb exposure produced sex‐specific changes in synaptic transmission and neuronal fiber excitability. Moreover, Pb exposure altered basal synaptic transmission and short‐term synaptic depression at vHPC–mPFC synapses, using stimulation frequencies relevant to working memory.

### 4.1 Effect of Pb exposure on synaptic transmission in CA1

Pb exposure during brain development results in high Pb levels in the hippocampus (Collins, Hrdina, Whittle, & Singhal, 1982) even long after exposure has ceased. Here, we examined how early Pb exposure affects CA1 synaptic transmission in mouse acute HPC slices. Our results show that early high‐level Pb did not affect synaptic transmission. This is similar to previous studies using male and female rats exposed to Pb prenatally until PND 21 (BLLs = 16.04–30.1 µg/dl), showing no effect on basal synaptic transmission or population spike amplitude in areas CA1, CA3, and dentate gyrus 30–45 days postexposure (Gutowski, Altmann, Sveinsson, & Wiegand, 1998; Xu et al., 1998; Zhao et al., 1999). Interestingly, the density of dendritic spines decreased in HPC neurons from rats continuously exposed to high‐level Pb from parturition to PND 30, 60, or 90 (BLLs = 21–38 µg/dl; Du et al., 2015). A decrease in spine density is typically associated with a decrease in synaptic transmission, which we did not observe in the mice exposed to high‐level Pb. This could be because our mice were only briefly (as opposed to continuously) exposed to Pb. Alternatively, a compensatory mechanism such as an increase in fiber excitability, in quantal content, or vesicular glutamate release (Braga, Pereira, Marchioro, & Albuquerque, 1999a) could have mitigated the Pb exposure effect on synaptic transmission.

We are the first to quantify synaptic transmission in the mouse CA1 following lowest level Pb exposure (yielding BLLs <15 µg/dl) known to alter cognitive processes. In fact, our data show that 8 months following low Pb exposure, synaptic transmission was decreased in CA1. This could be explained by a decrease in fiber excitability, or evoked‐glutamate release, as was previously shown in rat HPC neurons in culture (Braga, Pereira, & Albuquerque, 1999b) perfused with Pb concentrations lower than 2.1 µg/dl. Synaptic GABA release was also affected by such low Pb treatment. Whether early low‐level Pb exposure affects other neurotransmitters within the HPC region should be determined.

Previous reports on daily acclimation and handling (often used before behavioral assays) can cause alterations in sleep, stress, and levels of *N*‐methyl‐d‐aspartate receptor subunits. In our study, the mice used were only minimally manipulated not only at the time of Pb exposure prior to behavioral testing (PND 0 to PND 28), but also at the time of the electrophysiological slice experiments, 8–11 months following Pb exposure. Similar manipulation and handling were previously reported not to alter HPC synaptic transmission and plasticity in C57BL/6J mice (Vecsey et al., 2013). Therefore, we conclude that this handling is not likely to have impact on the synaptic results described here.

### 4.2 Effect of Pb exposure on CA3 fiber excitability

To further understand the mechanisms underlying the synaptic results above, we then assessed neuronal fiber excitability at CA3–CA1 synapses. Surprisingly, low Pb exposure did not affect presynaptic axonal response. In contrast, high Pb exposure significantly increased the amplitude of the fiber volley. Previous HPC slice studies support our high Pb exposure effects. First, in 60‐day‐old rats exposed to high‐level Pb from gestation until PND 21, an increased fEPSP amplitude was recorded in CA3 neurons (Xu et al., 1998). Also, in rat HPC slices, the perfusion of 5 µM of Pb (1,035 ppm) increased the action potential firing and decreased the spike frequency adaptation of pyramidal CA1 neurons (Yan et al., 2008). These effects were associated with alterations in intracellular Ca2+ flux through T‐type calcium channels (Yan et al., 2008). Our fiber excitability results could be due to similar synaptic and Ca‐dependent mechanisms.

Overall, our data suggest that early high‐level Pb exposure is associated with an increase in fiber excitability that could have mitigated a change in synaptic transmission. In contrast, although early lowest level Pb exposure did not change presynaptic neuronal fiber activity, it decreased synaptic transmission. Alternatively, the effects of both Pb levels could be caused by changes in presynaptic efficacy (e.g., glutamate release) and/or changes in postsynaptic mechanisms (e.g. postsynaptic receptor expression, activation, and distribution).

### 4.3 Consequences of Pb exposure on hippocampal short‐term synaptic plasticity

We then focused on synaptic plasticity which reflects a change in the efficacy of synaptic transmission. Facilitation is the presynaptic enhancement occurring within hundreds of milliseconds, which involves an increase in the number of transmitter quanta released by an AP without any change in quantal size or postsynaptic effectiveness (Zucker & Regehr, 2002). We used a paired‐pulse facilitation (PPF) protocol to investigate presynaptic enhancement of neurotransmitter release. Our results show no effect of Pb on synaptic facilitation in CA1. This is similar to 3‐month‐old male rats exposed to high‐level Pb from gestation until PND 21 (Gutowski et al., 1998), but in contrast to 50–day–old rats that were continuously exposed to high‐level Pb from the gestation period until the day of the experiment (Zhang et al., 2015). The different PPF results obtained between these studies could lie in the age at which the animals were tested: PND 50 (Zhang et al., 2015) versus PND > 90 (Gutowski et al., 1998 and our study). In fact, our data could reflect the long‐term restoration of presynaptic mechanisms back to normal state, a compensatory effect that can occur even under continuous Pb exposure, given enough time. As published using rats continuously exposed to high‐level Pb from birth to the experimental day 30, 60, or 90 (Du et al., 2015), pyramidal neurons exhibited some cytoarchitectural changes that were obvious at PND 30, but then absent at PND 60 and PND 90. Since PPR was not affected, changes we observed in basal neurotransmission detected could result from postsynaptic mechanisms (e.g., NMDA receptor expression, activation, and distribution). Alternatively, it is possible that Pb exposure induced long‐term presynaptic changes (such as changes in vesicular content) not revealed using a paired‐pulse paradigm. The absence of long‐term effect on PPF was observed under both Pb exposure levels. However, we are the first to show no alteration of PPF by early lowest level Pb exposure.

In addition to facilitation, many synapses display a decrease in synaptic strength with repetitive activation. In fact, at high stimulation frequencies, HPC synapses can undergo short‐term synaptic depression which contributes to overall network activity. Our results show that early Pb exposure did not alter STD at CA3–CA1 synapses. Previous work showed that LTD was decreased in 3‐month‐old rats exposed to high‐level Pb from birth to 21 (Zhao et al., 1999). STD and LTD differ in their underlying cellular mechanisms: STD only involves presynaptic mechanisms, whereas pre‐ and postsynaptic mechanisms contribute to LTD. While we did not assess LTD in our mice, it is tempting to speculate that both high and low Pb level exposures might affect more significantly postsynaptic mechanisms, since our experimental conditions did not alter STD within CA1.

Future experiments should be done to determine whether early chronic Pb exposure has long term effects on different forms of synaptic plasticity (short‐term and long‐term) in the HPC subregions. This is because at many synapses, the summation of these time‐dependent mechanisms shapes the overall synaptic strength (Zucker & Regehr, 2002).

### 4.4 Effects of early chronic Pb exposure at vHPC–mPFC synapses

While working memory declines with age (Aggleton, Blindt, & Candy, 1989; deToledo‐Morell, Geinisman, & Morrell, 1988; Ingram, London, & Goodrick, 1981), childhood Pb exposure increases the occurrence of working memory deficits in aging individuals (Cecil et al., 2008; Mazumdar et al., 2011; Schwartz BS et al., 2000). Working memory depends on the integrity of the frontohippocampal glutamatergic connection (Jay et al., 1992, 1995; Jones & Wilson, 2005b). Moreover, the strength of hippocampal–prefrontal synchrony is correlated with behavioral performance, including at theta frequencies (Hyman, Zilli, Paley, & Hasselmo, 2010; Jones & Wilson, 2005a, 2005b; Sigurdsson, Stark, Karayiorgou, Gogos, & Gordon, 2010). This is because the phase of theta oscillations in the hippocampus modulates the firing of mPFC neurons, and the local field potentials in the two structures display coherence in the theta frequency range (Benchenane et al., 2010; Hyman et al., 2010; Jones & Wilson, 2005a, 2005b; Siapas, Lubenov, & Wilson, 2005; Sigurdsson et al., 2010; Sirota et al., 2008) during a working memory task. Therefore, we evaluated the consequence of developmental low‐level Pb exposure on a neural pathway crucial for working memory. We show that early lowest level Pb exposure decreases synaptic transmission at vHPC–mPFC synapses. Moreover, when tested at frequencies relevant to working memory, high‐level Pb exposure decreased STD. We therefore suggest that although early low‐ and high‐level Pb exposure disrupt different synaptic mechanisms, they both can affect the working memory neuronal circuitry of aging subjects.

### 4.5 Sex‐specific effects of early chronic high‐ and low‐level Pb exposure

Previous rodent studies reported sex‐related differences when HPC‐dependent learning and memory tasks (Anderson, Pothakos, & Schneider, 2012; Flores‐Montoya & Sobin, 2015; Flores‐Montoya et al., 2015), as well as patterns of gene expression in the mPFC and the hippocampus (Anderson, Mettil, & Schneider, 2013; Schneider, Anderson, Sonnenahalli, & Vadigepalli, 2011) were evaluated under low‐level Pb exposure. Moreover, in a mouse model of human gestational Pb exposure (Leasure et al., 2008), a male‐specific change in motor activity was observed when year‐old male and female mice were compared 1 year after the exposure to low Pb levels had stopped. It was further proposed that hormonal differences and dopaminergic neurotransmission could play a role in the low Pb‐induced sex differences. In our current study, the adult mice that we tested were previously used, as juveniles, to model neurobehavioral changes that occur in children exposed to high and lowest level Pb (Flores‐Montoya et al., 2015). This study showed that in young male mice, BLLs were linearly correlated with a decreased olfactory recognition memory. In contrast, among low Pb‐exposed females, memory performance was not linearly related to blood Pb (Flores‐Montoya et al., 2015). By grouping our CA1 electrophysiology data by sex, Pb exposure levels lead to different results. In the case of male mice, low Pb exposure decreased synaptic transmission and increased fiber excitability. Therefore, low Pb exposure in male affected presynaptic (e.g., fiber excitability and maybe other presynaptic calcium‐independent mechanisms) and maybe also postsynaptic (e.g., postsynaptic receptor density or activity) mechanisms. Interestingly, high Pb exposure did not have a significant effect neither on synaptic transmission nor on fiber excitability. In the case of female mice, low Pb levels did not affect synaptic transmission or fiber volley amplitude. In contrast, high Pb levels increased synaptic transmission, and it is tempting to speculate that this increase is due to the increase in fiber excitability caused by high Pb exposure in females. In sum, these results are in line with the olfactory memory assay described in Flores‐Montoya et al., 2015, where low Pb exposure affected the behavior of male mice without affecting the behavior of female mice.

At the end of our study, we ran out of low Pb‐exposed males and high Pb‐exposed females. In an attempt to compensate for that, the number of recorded HPC slices per animals was increased. The “*N*” number was also taken into account by the statistical analysis. Finally, our data showed modest variability, therefore, the small “*N*” number should not significantly impact the conclusion of our results.

Together, these findings suggest that depending on Pb levels, brief early exposure can lead to sex‐specific effects on the HPC neural circuits of adult subjects. Such findings should encourage lifetime assessment of the neurotoxic consequences of early Pb exposure especially in at risk populations.

## CONFLICT OF INTEREST

There was no conflict of interests stated by the authors.

## DATA AVAILABILITY STATEMENT

The data that support the findings of this study are available from the corresponding author upon reasonable request.
